# CMD: A Database to Store the Bonding States of Cysteine Motifs with Secondary Structures

**DOI:** 10.1155/2012/849830

**Published:** 2012-10-10

**Authors:** Hamed Bostan, Naomie Salim, Zeti Azura Hussein, Peter Klappa, Mohd Shahir Shamsir

**Affiliations:** ^1^Faculty of Biosciences and Bioengineering, Universiti Teknologi Malaysia, 81310 Johor Bahru, Johor, Malaysia; ^2^Faculty of Computer Science and Information Systems, Universiti Teknologi Malaysia, 81310 Johor Bahru, Johor, Malaysia; ^3^School of Bioscience and Biotechnology, Faculty of Science and Technology, Universiti Kebangsaan Malaysia, 43600 Bangi, Selangor, Malaysia; ^4^School of Biosciences, University of Kent, Canterbury, Kent CT2 7NJ, UK

## Abstract

Computational approaches to the disulphide bonding state and its connectivity pattern prediction are based on various descriptors. One descriptor is the amino acid sequence motifs flanking the cysteine residue motifs. Despite the existence of disulphide bonding information in many databases and applications, there is no complete reference and motif query available at the moment. Cysteine motif database (CMD) is the first online resource that stores all cysteine residues, their flanking motifs with their secondary structure, and propensity values assignment derived from the laboratory data. We extracted more than 3 million cysteine motifs from PDB and UniProt data, annotated with secondary structure assignment, propensity value assignment, and frequency of occurrence and coefficiency of their bonding status. Removal of redundancies generated 15875 unique flanking motifs that are always bonded and 41577 unique patterns that are always nonbonded. Queries are based on the protein ID, FASTA sequence, sequence motif, and secondary structure individually or in batch format using the provided APIs that allow remote users to query our database via third party software and/or high throughput screening/querying. The CMD offers extensive information about the bonded, free cysteine residues, and their motifs that allows in-depth characterization of the sequence motif composition.

## 1. Background

Disulphide bonds are formed by oxidation of two cysteine residues in a protein and are significant to a protein's conformational stability as they confer greater thermal and chemical stability as well as stabilizing structural intermediates to ensure the correct folding pathway. However, the connectivity of the disulphide bonds in protein sequences can only be determined experimentally. Given this difficulty, the ability to evaluate or predict the disulphide bonding state and connectivity from the sequence would prove to be highly valuable in engineering proteins for biotechnological and medical applications. Computational approaches towards disulphide connectivity prediction have been based on various descriptors. One of these descriptors is the sequence motifs generated by combining the flanking residues on the either side of the the cysteine residue [1, 2]. These immediate residues flanking the cysteine have been shown to influence the cysteine's redox potential and the cysteine's steric accessibility [3]. These sequence motifs have been fed into various prediction methods [4] such as machine learning approaches (i.e., statistical methods, neural networks (NNs) [5], and support vector machine (SVM) [6–8] such as DiaNNA [3], DISULFIND [9], DCON [10], and CysView [11]. Currently, all the cysteine motifs are extracted by parsing data from protein databases and feeding them into the prediction tools. Motivated by the absence of a database and usefulness of the cysteine flanking motifs in predicting the cysteine bonding state and connectivity prediction, we have developed cysteine motif database (CMD) as a tool to mine and store these motifs. The creation of CMD allows the motif extraction and facilitates the study of their secondary structures, bonding and connectivity propensities. In this paper, we present CMD as a publicly available tool that complements existing prediction tools.

## 2. Construction and Content

### 2.1. Content

The CMD data was compiled from Protein Data Bank (PDB) (http://www.rcsb.org) and UniProt (http://www.uniprot.org). For each databank, two different datasets were created; a complete protein dataset and a second 100% nonhomologous unique sequence dataset (100% similar sequences were omitted). We have featured CMD with both datasets for each PDB and UniProt, allowing researchers to utilize the database in its entirety (73656 structures for PDB and 531462 structures for UniProt) or to include only unique sequences (33874 for PDB and 140723 for UniProt). Using these datasets, we extracted 878,000 cysteine motifs based on 1st, 2nd, 3rd, 4th, and 5th flanking residues of the cysteine as these immediate residues are within proximity to exert influence on the cysteine (Table 1). The assignment of the bonding state of cysteine residues and their bonding partners is based on the SSBOND and DISULPHIDE BOND tags in each PDB and UniProt files. The motifs were clustered according to the occurrence of the bonding state, that is, always bonded, always nonbonded, and both bonded and nonbonded (nonbonded state with another cysteine or to other atoms such as metals). Each of the bonded cysteine is also mapped to each inter and intra-chain disulphide bond cysteine partner.

The motifs were categorized between inter and intradomain with the secondary structure assignments for each motif sequence (if available) determined using secondary structure reference files retrieved from PDB.

### 2.2. Construction

The data contained in CMD is stored in Microsoft SQL server 2005 data storage architecture. Cysteine motif pattern tables are indexed based on Protein ID, motifs, chain number, and secondary structure to enhance the efficiency of the querying performance. Table-based partitioning was used to increase the flexibility and performance on Motif data tables. In these tables, over three million motifs are stored which can be queried and processed. All preprocessing, data extraction, and injection for motif sequences and their secondary structure were carried out in Net 4.0 platform using C# programming language. The web interface of CMD is based on ASP. Net extension integrated with Ajax technology to provide a strong, simple, and user friendly environment for end users. The web application is hosted on an Internet Information Services (IIS) HTTP server version 7.5.7600.16385. CMD will be updated automatically with latest data from PDB and Uniprot.

In addition, several APIs available in CMD enable developers to query our database remotely and embed the results in their own system independently. A complete list of available APIs together with the method of inline implementation is available in the FAQ section of the CMD website.

## 3. Data Update

Using RCSB and UniProt API's, the software will retrieve all the Protein IDs available in the mentioned resources. A query will list all the existing Protein IDs in our local dataset. All new Protein IDs will be identified using both above references. Using RCSB and UniProt ftp services, all the newly identified protein files will be downloaded using the Protein ID's to our local server. As in our method of preprocessing and data set preparation, all SEQRESS and SSBOND tags will be extracted from the downloaded files. All cysteine motifs based on the 1st, 2nd, 3rd, 4th, and 5th number of flanking residue on each side (neighboring residues) will be captured and extracted to the records of data with cysteine at the meddle. Each record contains the motif sequence, Chain ID, cysteine residue position in the sequence, bonding status of cysteine residue and the Protein ID as the reference. Each record will be inserted into our database. A log will be generated for the successful procedure or any run time error.

## 4. Utility and Discussion

### 4.1. User Interface

**Figure 1:**
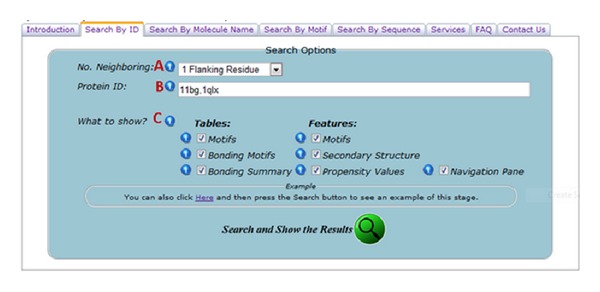
Annotated diagram describing the search options for “Search By ID” section. (A) Users can choose either PDB or SwissProt. (B) Users can enter single or multiple ProteinIDs separated by comma (,) as keyword. (C) Users can choose which of the results to appear in the output.

**Figure 2:**
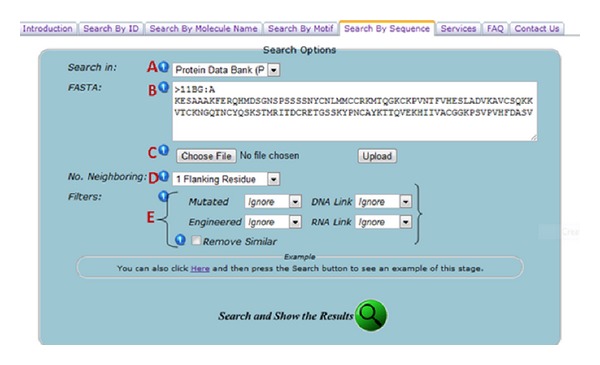
Annotated diagram of “Search By FASTA Sequence” section showing all search options and filtering criteria. (A) Users can choose either PDB or SwissProt. (B) Users can enter single or multiple FASTA sequences to be investigated for each motif inside. (C) Users can also upload a FASTA format file to be investigated. (D) Users can choose the number of amino acid residues on each side of cysteine for motif extraction process within the FASTA sequence. (E) Users can filter the proteins in which the motif will be investigated. User can specify whether the protein was engineered or mutated and choose whether the protein contains any DNA or RNA link. They can also filter out the similar proteins and keep only one identical copy of them for advanced investigations.

**Figure 3:**
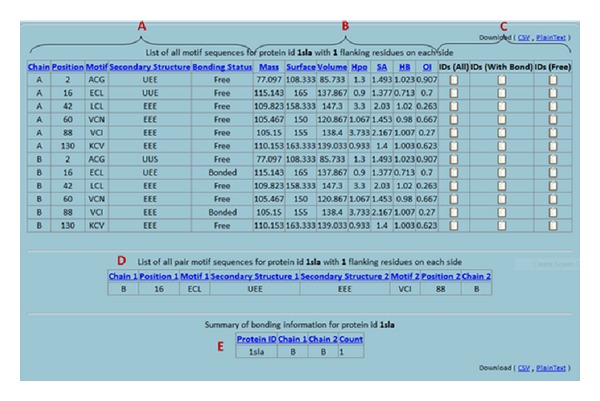
Annotated diagram describing the result's annotation for the “Search By Molecule Name” section. (A) Showing the motifs, secondary structure, cysteine position in the sequence, and the chain name. (B) Showing the propensity values of the motif sequence. (C) The navigation pane facilitating accessing ProteinIDs having common and similar features. (D) Listing the pair patterns existing in the protein in details. (E) The summary of bonding for the selected protein.

The CMD website features an interactive and comprehensive cysteine Motif query engine by supporting different search keywords, such as Protein IDs and motif sequences in the FASTA format. Users can filter according to proteins which are mutated and engineered proteins. All results can be downloaded as text and CSV for further analysis (Figures 1, 2, and 3).

### 4.2. Utility: Example Applications

CMD facilitate studies focused on cysteine disulphide bonding status prediction and analysis by processing the data. Here we present two applications of our system that illustrate the potential of CMD in greater details.

#### 4.2.1. Application 1: Statistical Analysis of Bonding State

To analyze the predictive power of CFMD, we investigated the cysteine bonding pattern of human protein disulphide isomerase (PDII, P07237 [UniParc]). PDI catalyses the formation (oxidation) and rearrangement (isomerisation) of disulphide bonds during the folding of secretory and membrane-bound proteins (for review see [12]), thus stabilising the native structure of these proteins. PDI contains two domains with high sequence homology to thioredoxin. One of these thioredoxin motives is found at position 52–55, while the second motif is located at position 396–399. The active site cysteine residues in the thioredoxin motives are essential for the oxidase/isomerase activity of PDI. In each motif the two cysteine residues within the sequence—WCGHC—can potentially form a disulphide bond.

**Figure 4:**
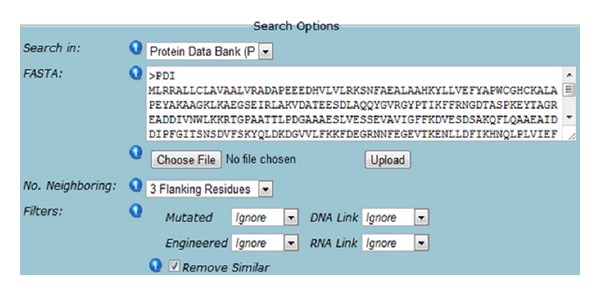
Query for full length human protein disulphide isomerase (PDII, P07237 [UniParc]). (A) Screenshot of parameters for CFMD.

To investigate whether both thioredoxin motives have similar disulphide bond propensities, that is, whether both thioredoxin motives are in the same bonded form, we analysed the disulphide bonding pattern with the CFMD (Figure 4 and Table 2). Our analysis predicted that the first thioredoxin motif around residues 52–55 indeed forms an intradomain disulphide bond; the second cysteine residue in the sequence CGHCKAL has a very high propensity of forming a disulphide bond with the first cysteine residue. However, the second thioredoxin motif is not predicted to be disulphide bonded, since the second cysteine residue in the sequence CGHCKQL has zero propensity of forming a disulphide bond with the first cysteine residue in this motif. We therefore predict that the two thioredoxin motives in PDI are in different bonding states; while the first—WCGHC—motif is in the oxidized and thus disulphide bonded form, the second thioredoxin motif is in the reduced form. From this analysis we conclude that the two thioredoxin motives in PDI have different reduction potentials. This result is in excellent agreement with the findings of Chambers and co-workers [13], who showed that the two thioredoxin motives react differently to Ero1a, the *in vivo* oxidant of PDI.

#### 4.2.2. Application 2: Protein Identification and Motif Exploration

Catalytic functionalities of some enzymatic proteins are dependant on the oxidation and reduction of state of their cysteine residues. The oxidation of cysteine residues and formation of disulphide bonds take place in a reducing environment. In prokaryotes, disulphide bonds are mainly formed in the periplasmic space outside the membrane. In contrast, the formation of disulphide bonds takes place in endoplasmic reticulum (ER) in eukaryotes. As a result, proteins with stable disulfide bonds rarely reside in the cytoplasm. This knowledge would apply on a larger scale, making the local and global profile of each protein environment, its folding localization, and classification becoming a potential contribution on the disulphide bonding prediction mechanism.

CMD offers the user a unique ability to identify and mine all known proteins using specific motif sequence, and explore their classification, motif sequences, structure, and bonding status. During the creation of the datasets, we discovered 15875 unique motifs that are always bonded (EATLRCWALGF with the highest occurrence) and 41577 unique patterns that are always nonbonded (ALSVPCSDSKA with the highest occurrence) for the five flanking residues that can be utilized for cysteine state prediction. The number of these unique motifs is considerably higher than prior number of motifs used in cysteine bond prediction [3, 14] and not limited to specific genomes [15].

### 4.3. Data Availability

The CMD databases are accessible through a web portal at http://birg4.fbb.utm.my/cmd. The entire database with annotations is available for download in the SQL format, describing the relations between classes and fragments. As an additional service for programmers and third party developers, all queries available in CMD are freely accessible using available web services and web application programming interfaces (API). Also for automated high-throughput querying, all information contained in the CMD database can be downloaded using ftp services.

## 5. Discussion

The CMD combined data of bonded and free cysteine motifs aims to fill a gap in the knowledge query that will allow in-depth characterization of the composition propensity, and its role in determining the bonding state. Despite the bonding information regarding cysteine residues in proteins available in many databases and several applications focused on disulphide bridge formation prediction, there is no complete reference with a proper form of representation and analysis available at the moment. This database is automatically updated from the PDB and UniProt that currently contain 878000 cysteine motifs with more than 77,000 unique cysteine motifs and cysteine pairing motifs. Compilation of these cysteine motifs together with their secondary structures and propensity value assignments, and the ability to query using Protein IDs and motif sequences is a novel and significant feature over prior prediction works which use considerably smaller datasets [3]. In addition to the novelty of the motif query tool, CMD has several novelties such as inclusion of UniProt data, the distinction between inter or intrachain disulphide bonds, inter or intradomain bonds, and an application programming interfaces (APIs) for interfacing with other bioinformatics tools.

## 6. Conclusion

The creation of CMD is useful when analyzing cysteine/disulfide bond formation and its motif sequence composition analysis by providing (1) a query tool for cysteine motifs based upon a comprehensive cysteine motif database curated from PDB and UniProt, (2) secondary structure and propensity values assignments of each motif sequence, and (3) datasets of detailed information of the motifs such as occurrence frequency and their amino acids propensity value. We believe that CMD's usefulness will be the query tool that will complement other protein 3D structural databases and similarly motif-based prediction tools.

## Figures and Tables

**Table 1:** 

	PDB (All)	PDB (NH)	UniProt (All)	UniProt (NH)
Proteins	73656	33874	531462	140723
Patterns	535544	230213	2509611	966374
Bonded motifs	148505	64246	189238	113365
Nonbonded motifs	387039	165967	2320373	853009
Intrachain	84591	36473	—	—
Interchain	4013	1900	—	—

NH: Nonhomologous unique sequences which have been affected by 100% similarity removal.

**Table 2:** Edited output from (A). The bold rows indicate the second active site cysteine residues in the respective thioredoxin motif. Column 1 (Thioredoxin motif) was added for additional clarification. The cysteine residue in italics indicates the queried cysteine residue, the respective position of which is given in the second column.

Thioredoxin motif	Position	Motif	Total	Bond	Coefficient
			0	0	0
1	52	APW*C*GHC	12	5	0.417
**1**	**55**	**CGH*C*KAL**	**1**	**1**	**1**
			0	0	0
			0	0	0
2	396	APW*C*GHC	12	5	0.417
**2**	**399**	**CGH*C*KQL**	**2**	**0**	**0**
